# Evaluation of Serum Soluble Urokinase Plasminogen Activator Receptor (suPAR) Levels in Different Metabolic Obesity Phenotypes and Their Association with Inflammatory Cytokines: A Cross-Sectional Study

**DOI:** 10.3390/jcm15186990

**Published:** 2026-09-09

**Authors:** Aycan Acet, Cagla Ozdemir, Hatice Solak

**Affiliations:** 1 Department of Internal Medicine, Faculty of Medicine, Kutahya Health Sciences University, 43100 Kutahya, Türkiye; 2 Department of Family Medicine, Faculty of Medicine, Kutahya Health Sciences University, 43100 Kutahya, Türkiye; cagla.ozdemir@ksbu.edu.tr; 3 Department of Physiology, Faculty of Medicine, Kutahya Health Sciences University, 43100 Kutahya, Türkiye; hatice.solak@ksbu.edu.tr

**Keywords:** insulin resistance, metabolic obesity phenotypes, suPAR, CXCL10, chronic inflammation

## Abstract

**Background:** The classification of metabolic risk solely based on body mass index (BMI) fails to acknowledge the heterogeneity inherent in obesity. Soluble urokinase plasminogen activator receptor (suPAR) has been proposed as a stable biomarker of chronic inflammation; however, its behaviour across metabolic obesity phenotypes defined jointly by adiposity and insulin resistance remains uncertain. **Methods:** In this cross-sectional study, 190 adults aged 18 to 60 years attending internal medicine outpatient clinics were stratified by BMI (≥30 kg/m^2^) and homeostasis model assessment of insulin resistance (HOMA-IR ≥ 2.5) into four phenotypes: non-obese insulin-sensitive (NOIS, *n* = 64), non-obese insulin-resistant (NOIR, *n* = 50), obese insulin-sensitive (OIS, *n* = 33), and obese insulin-resistant (OIR, *n* = 43). Serum suPAR, interleukin-6 (IL-6), and C-X-C motif chemokine ligand 10 (CXCL10) were measured using enzyme-linked immunosorbent assays. Group comparisons, rank-based two-factor analyses, correlation analyses, logistic regression, and receiver operating characteristic (ROC) analyses were performed. **Results:** Serum suPAR did not differ across the four phenotypes (*p* = 0.19) and was not correlated with HOMA-IR (ρ = −0.09, *p* = 0.24). IL-6 was lower in the OIR group than in the NOIS group (*p* = 0.012). CXCL10 was higher in both obese phenotypes than in both non-obese phenotypes (*p* < 0.001), and rank-based two-factor analysis attributed this difference to obesity (*p* < 0.001) rather than insulin resistance (*p* = 0.71). CXCL10 correlated with BMI (ρ = 0.29, *p* < 0.001) and remained associated with obesity after adjustment for age, sex, and C-reactive protein (odds ratio per 10 ng/L, 1.12; 95% confidence interval, 1.06 to 1.18). CXCL10 discriminated obesity with an area under the ROC curve of 0.74 (95% CI, 0.66 to 0.81). None of the three biomarkers discriminated insulin resistance (areas under the curve, 0.43 to 0.50). The association between CXCL10 and obesity was consistent in both sexes and unchanged with alternative HOMA-IR cutoffs of 2.0 and 3.0. **Conclusions:** In this cohort, circulating CXCL10 tracked adiposity rather than insulin resistance, whereas suPAR and IL-6 did not separate metabolic obesity phenotypes. These findings do not support suPAR as a marker of insulin resistance independently of body fat.

## 1. Introduction

Obesity now affects more than 1 billion people worldwide, and its prevalence has continued to rise in most countries over the past three decades [1]. Excess adiposity promotes a state of chronic low-grade inflammation, in which adipose tissue dysfunction, immune cell infiltration, and cytokine release contribute to insulin resistance and the development of type 2 diabetes mellitus (T2DM) and cardiovascular disease [2]. Nevertheless, the metabolic consequences of obesity are heterogeneous. A subset of individuals with a body mass index (BMI) of 30 kg/m^2^ or higher preserves insulin sensitivity and a favourable metabolic profile, a state described as metabolically healthy obesity, whereas some normal-weight individuals harbour insulin resistance and subclinical inflammation [3,4]. Because even metabolically healthy obesity carries an excess long-term cardiovascular risk, as shown in a meta-analysis of more than 4.8 million participants, refining risk assessment beyond BMI has become a priority [5].

The soluble urokinase plasminogen activator receptor (suPAR) is released from immunologically active cells, circulates at stable concentrations without circadian variation, and has been proposed as an integrative index of systemic chronic inflammation [6,7]. In general population cohorts, higher suPAR concentrations predict incident T2DM, cardiovascular events, and mortality independently of C-reactive protein (CRP) [8,9]. The relationship between suPAR and body weight appears to be complex. In a Danish cohort of individuals with impaired glucose regulation, suPAR predicted incident T2DM among overweight participants but not among obese participants [10], and in healthy adults only a modest independent association between suPAR and body weight has been reported [11]. Whether suPAR reflects insulin resistance independently of adiposity remains unresolved.

C-X-C motif chemokine ligand 10 (CXCL10), an interferon-inducible chemokine that recruits CXCR3-positive lymphocytes, participates in a broad range of inflammatory conditions [12]. Human adipocytes constitutively secrete CXCL10 [13], and adipose tissue expression of CXCL10 is increased in obesity and correlates with BMI [14]. Interleukin-6 (IL-6), a canonical adipose-derived cytokine, increases with expanded fat mass [15]. Data on the joint behaviour of these mediators across phenotypes defined simultaneously by adiposity and insulin resistance are limited.

The present study aimed to compare serum suPAR, IL-6, and CXCL10 concentrations across four metabolic obesity phenotypes defined by body mass index (BMI) and homeostasis model assessment of insulin resistance (HOMA-IR), and to examine the relationship between these biomarkers and anthropometric and metabolic parameters.

## 2. Materials and Methods

### 2.1. Study Design and Participants

This cross-sectional study was conducted at the internal medicine outpatient clinics of Kütahya City Hospital, a tertiary-care centre, between February and May 2026. Consecutive volunteers aged 18 to 60 years were enrolled if they attended for a routine health assessment or for symptoms suggestive of insulin resistance or hyperglycaemia. Because metabolic phenotyping required a complete fasting panel, only individuals with available measurements of fasting glucose, insulin, lipid profile, creatinine, aminotransferases, uric acid, CRP, complete blood count, and haemoglobin A1c (HbA1c) obtained as part of routine care were included. The exclusion criteria were active infection (CRP > 5 mg/L), a history of diabetes, malignancy, or autoimmune disease, chronic cardiac, hepatic, or renal disease, obstructive pulmonary disease, chronic inflammatory bowel disease, current use of steroidal or nonsteroidal anti-inflammatory drugs, alcohol or substance use, surgery within the preceding three months, and pregnancy or lactation. All participants provided written informed consent after reading the study information form, and the study was conducted in accordance with the Declaration of Helsinki (Institutional Ethics Committee approval no. 2026/01-34).

### 2.2. Phenotype Definitions

BMI was calculated as weight in kilograms divided by the square of height in meters, and obesity was defined as a BMI of ≥30 kg/m^2^ according to the World Health Organization criteria [16]. Waist circumference was measured using a non-elastic tape, and weight was measured using a calibrated digital scale under standard outpatient conditions. Insulin resistance was quantified using HOMA-IR, calculated as fasting insulin (µU/mL) multiplied by fasting glucose (mg/dL) divided by 405 [17], with a cutoff of 2.5 [18].

Participants were assigned to four phenotypes: non-obese insulin-sensitive (NOIS; BMI < 30 kg/m^2^, HOMA-IR < 2.5), non-obese insulin-resistant (NOIR; BMI < 30 kg/m^2^, HOMA-IR ≥ 2.5), obese insulin-sensitive (OIS; BMI ≥ 30 kg/m^2^, HOMA-IR < 2.5), and obese insulin-resistant (OIR; BMI ≥ 30 kg/m^2^, HOMA-IR ≥ 2.5).

### 2.3. Laboratory Measurements

After an overnight fast, approximately 10 mL of venous blood was drawn from the antecubital vein into gel separator tubes and centrifuged at 4000 rpm for 10 min. Serum samples were divided into three aliquots in Eppendorf tubes and stored at −20 °C until analysis. After completion of sample collection, serum suPAR (Cat. No. E3759Hu), CXCL10 (Cat. No. E3800Hu), and IL-6 (Cat. No. E0090Hu) levels were measured using commercially available ELISA kits (BT LAB, Shanghai, China) according to the manufacturer’s instructions. Results for all three biomarkers were expressed in ng/L. For suPAR, the manufacturer-reported analytical sensitivity was 3.82 ng/L, with a measurement range of 5–1900 ng/L; the reported intra-assay and inter-assay coefficients of variation were <8% and <10%, respectively. Routine biochemical and hematological parameters were retrieved from the hospital information system.

### 2.4. Statistical Analysis

Sample size adequacy was assessed for the primary comparison of biomarker concentrations across the four phenotypes. In a previous case–control study on obesity, circulating inflammatory markers discriminated obese from non-obese participants with areas under the curve of 0.63 to 0.88, corresponding to standardised differences of approximately 0.45 to 1.68 [19]. Therefore, a medium effect size (Cohen’s f = 0.25) was assumed for the four-group comparison. With a two-sided α of 0.05 and 80% power, 179 participants were required, or 187 after allowance for the asymptotic relative efficiency of the Kruskal–Wallis test relative to analysis of variance (0.955). The available sample of 190 participants met this requirement and provided 81% power for the primary analysis. Distributional assumptions were examined using the Shapiro–Wilk test within each phenotype. Because most variables departed from normality, continuous data are summarised as medians with interquartile ranges (IQRs) and categorical data as counts with percentages. Differences across the four phenotypes were tested using the Kruskal–Wallis test, followed by Dunn post hoc comparisons with Bonferroni correction; the chi-square test was used for categorical variables. To separate the contributions of obesity and insulin resistance, a rank-based two-factor analysis of variance (Scheirer–Ray–Hare test) was performed with obesity status, insulin resistance status, and their interaction as factors. Associations between biomarkers and metabolic parameters were assessed with Spearman correlation, and rank-based partial correlations were computed with adjustment for BMI or HOMA-IR together with age and sex. The independent association of CXCL10 with obesity was evaluated with multivariable logistic regression adjusted for age, sex, and CRP. Discriminative performance was quantified with receiver operating characteristic (ROC) analysis; areas under the curve (AUC) are reported with 95% confidence intervals (CI), and optimal cutoffs were derived with the Youden index. In an additional subgroup analysis, serum CXCL10 concentrations were compared across World Health Organization obesity classes (non-obese, BMI < 30.0 kg/m^2^, class I, 30.0 to 34.9 kg/m^2^, class II, 35.0 to 39.9 kg/m^2^, class III, ≥40.0 kg/m^2^) with the Kruskal–Wallis test and Dunn post hoc comparisons, and the presence of an ordered trend across BMI categories was assessed with the Jonckheere–Terpstra test. To account for the predominance of women in the sample, biomarker concentrations were compared between sexes, the phenotype comparisons were repeated within each sex, and rank-based two-factor models with obesity status, sex, and their interaction were fitted. In sensitivity analyses, the rank-based obesity and insulin resistance models were re-estimated with alternative HOMA-IR cutoffs of 2.0 and 3.0, and HOMA-IR was additionally examined as a continuous variable. The waist-to-height ratio was calculated as waist circumference divided by height, and its associations with the biomarkers were assessed with Spearman and rank-based partial correlations. Two-tailed *p* values below 0.05 were considered to indicate statistical significance. Analyses were performed using IBM SPSS Statistics for macOS, version 31.0 (IBM Corp., Armonk, NY, USA) and Python (Python Software Foundation, Wilmington, DE, USA, version 3.12, SciPy and statsmodels).

## 3. Results

### 3.1. Characteristics of the Study Population

A total of 190 participants were included: 64 in the NOIS group, 50 in the NOIR group, 33 in the OIS group, and 43 in the OIR group. Overall, 138 participants (72.6%) were women, and the sex distribution was similar across the phenotypes (*p* = 0.95). Participants with obese phenotypes were older than those in the NOIS group (*p* = 0.005). By design, BMI, waist circumference, fasting insulin, and HOMA-IR differed markedly among the phenotypes (*p* < 0.001). Fasting glucose was higher in insulin-resistant phenotypes, whereas HbA1c, triglycerides, low-density lipoprotein cholesterol, uric acid, and alanine aminotransferase increased, and high-density lipoprotein cholesterol decreased along the phenotype gradient. CRP increased stepwise from the NOIS to the OIR group and was the highest in the OIR group. The baseline characteristics of the study population are shown in Table 1.

### 3.2. Inflammatory Biomarkers Across Phenotypes

**Figure 1:**
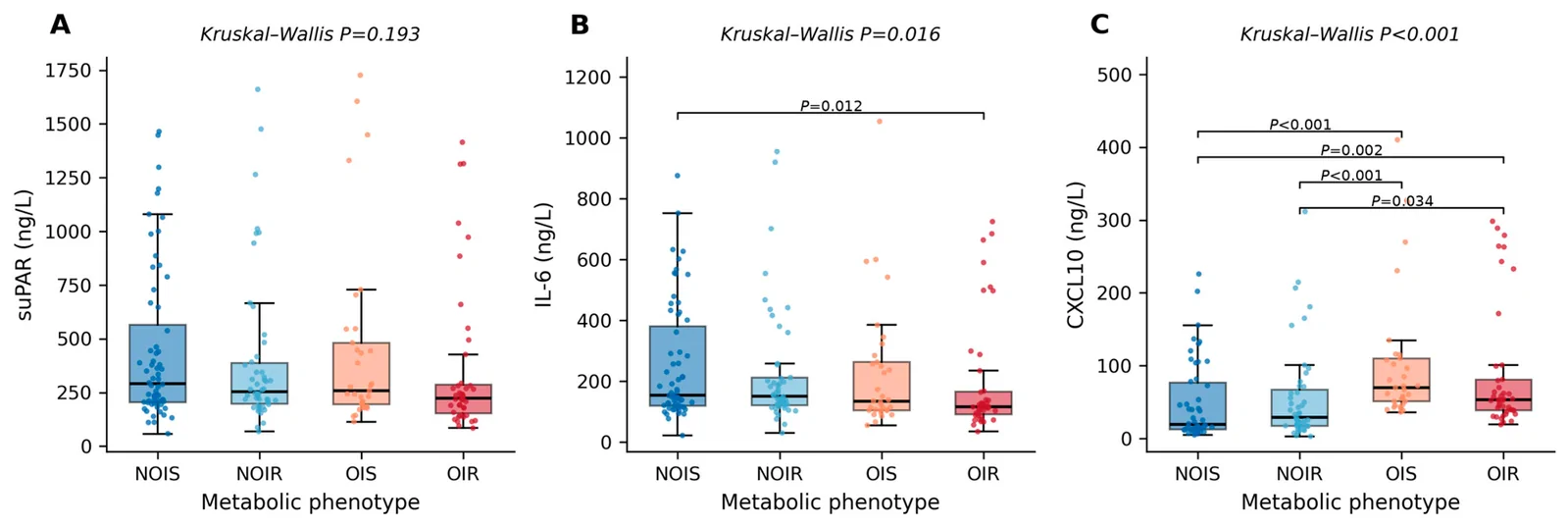
Serum suPAR (Panel (**A**)), IL-6 (Panel (**B**)), and CXCL10 (Panel (**C**)) concentrations in the four metabolic obesity phenotypes. Boxes indicate the medians and interquartile ranges, whiskers extend to 1.5 times the interquartile range, and dots represent individual participants. *p* values above the panels are from Kruskal–Wallis tests; brackets show Dunn’s post hoc comparisons with Bonferroni adjustment.

Serum suPAR concentrations did not significantly differ across the four phenotypes (Kruskal–Wallis *p* = 0.19), with the lowest median value numerically observed in the OIR group (Table 2 and Figure 1A). IL-6 levels differed modestly across phenotypes (*p* = 0.016); in post hoc comparisons, the OIR group had lower IL-6 levels than the NOIS group (adjusted *p* = 0.012) (Figure 1B). CXCL10 showed the clearest separation (*p* < 0.001); concentrations in both obese phenotypes exceeded those in both non-obese phenotypes (adjusted *p* ≤ 0.034 for all four obese versus non-obese contrasts), whereas the OIS and OIR groups did not differ from each other, and neither did the NOIS and NOIR groups (Figure 1C). The median CXCL10 level was 58.1 ng/L (IQR, 42.9 to 101.4) in obese participants and 25.4 ng/L (IQR, 13.8 to 72.1) in non-obese participants (*p* < 0.001).

Rank-based two-factor analysis assigned the variation in CXCL10 to obesity (*p* < 0.001), with no independent effect of insulin resistance (*p* = 0.71) and no interaction (*p* = 0.12). A main effect of obesity in the opposite direction was detected for IL-6 (*p* = 0.008), again without an effect of insulin resistance (*p* = 0.18). For suPAR, neither factor reached significance (obesity, *p* = 0.24; insulin resistance, *p* = 0.13).

### 3.3. Correlation Analyses

**Figure 2:**
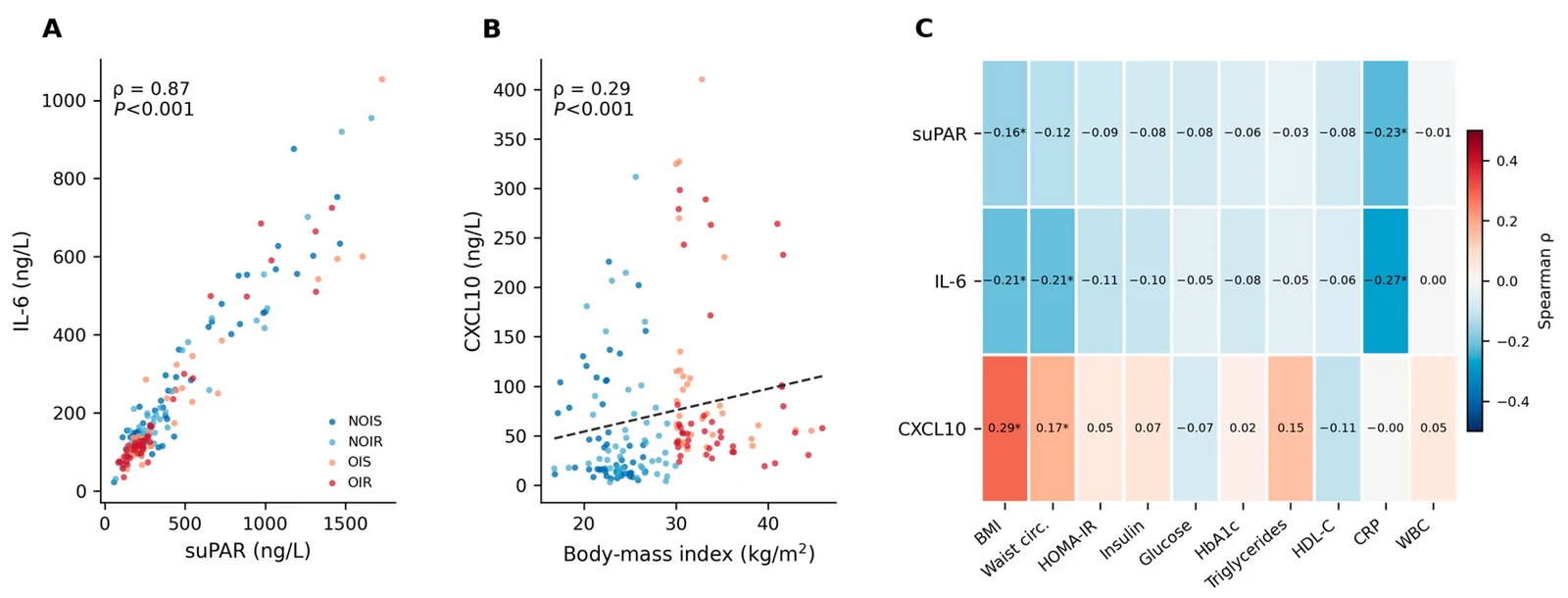
Correlation structure of inflammatory biomarkers. Panel (**A**) shows the relation between serum suPAR and IL-6 (Spearman ρ = 0.87) with participants coloured by phenotype. Panel (**B**) shows the relation between body mass index and CXCL10 (ρ = 0.29); the dashed line is a linear fit. Panel (**C**) shows the Spearman correlation coefficients between each biomarker and metabolic parameters; asterisks indicate *p* < 0.05.

The three ELISA-measured biomarkers were strongly intercorrelated (suPAR and IL-6, ρ = 0.87; suPAR and CXCL10, ρ = 0.66; IL-6 and CXCL10, ρ = 0.57; *p* < 0.001 for each), and these intercorrelations were consistent within each phenotype (Figure 2A). suPAR was not correlated with HOMA-IR (ρ = −0.09, *p* = 0.24), fasting insulin, glucose, HbA1c, or lipid parameters, and showed weak inverse correlations with BMI (ρ = −0.16, *p* = 0.027) and CRP (ρ = −0.23, *p* = 0.002) levels. IL-6 followed a similar inverse pattern to BMI, waist circumference, and CRP. In contrast, CXCL10 levels correlated positively with BMI (ρ = 0.29, *p* < 0.001) and waist circumference (ρ = 0.17, *p* = 0.022) but not with HOMA-IR (ρ = 0.05, *p* = 0.51) (Figure 2B,C). In rank-based partial correlation analyses, the association between CXCL10 and BMI persisted after adjustment for HOMA-IR, age, and sex (partial r = 0.30, *p* < 0.001), whereas no biomarker was associated with HOMA-IR after adjustment for BMI, age, and sex. CXCL10 also correlated positively with the waist-to-height ratio (ρ = 0.18, *p* = 0.016), and this association persisted after adjustment for HOMA-IR, age, and sex (partial r = 0.20, *p* = 0.007), whereas suPAR (ρ = −0.15, *p* = 0.044) and IL-6 (ρ = −0.24, *p* < 0.001) showed weak inverse correlations with this index, mirroring their inverse relations with BMI.

### 3.4. Discriminative Performance

**Figure 3:**
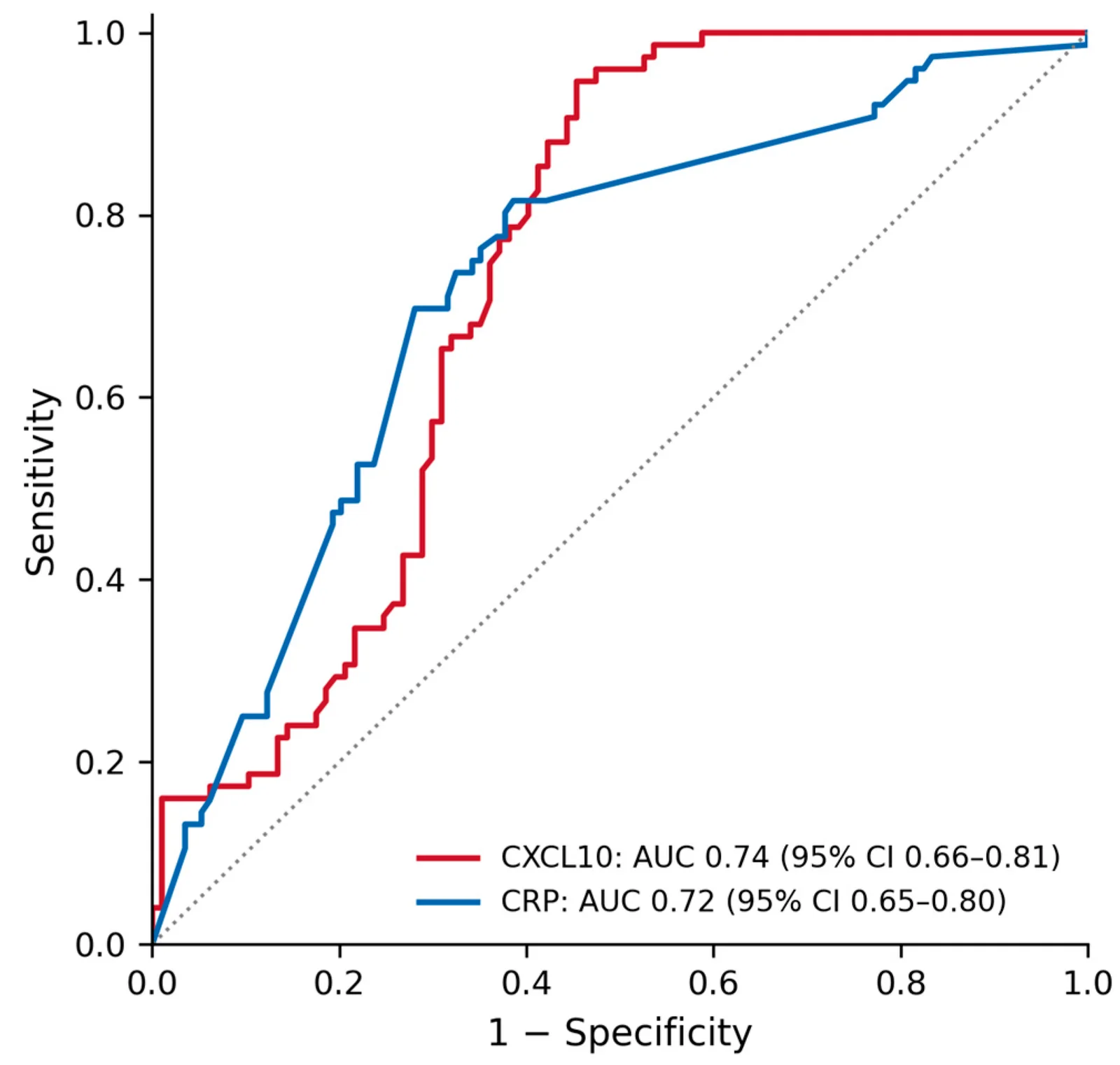
Receiver operating characteristic curves for CXCL10 and CRP in discriminating obesity (body mass index ≥ 30 kg/m^2^). The dotted diagonal line indicates no discrimination.

CXCL10 discriminated obese from non-obese participants with an AUC of 0.74 (95% CI, 0.66 to 0.81), comparable to CRP (AUC, 0.72; 95% CI, 0.65 to 0.80) (Figure 3). The Youden-optimal CXCL10 cutoff of 29.6 ng/L yielded a sensitivity of 94.7% and a specificity of 54.6% for obesity. In multivariable logistic regression, each 10 ng/L increase in CXCL10 was associated with obesity independently of age, sex, and CRP (odds ratio, 1.12; 95% CI, 1.06 to 1.18; *p* < 0.001); CRP retained an independent association in the same model (odds ratio, 1.87 per mg/L; 95% CI, 1.42 to 2.47). None of the three biomarkers discriminated insulin resistance defined by HOMA-IR of 2.5 or higher (AUC: suPAR, 0.43; IL-6, 0.43; CXCL10, 0.50). The diagnostic indices are summarised in Table 3.

### 3.5. CXCL10 According to Obesity Class

**Figure 4:**
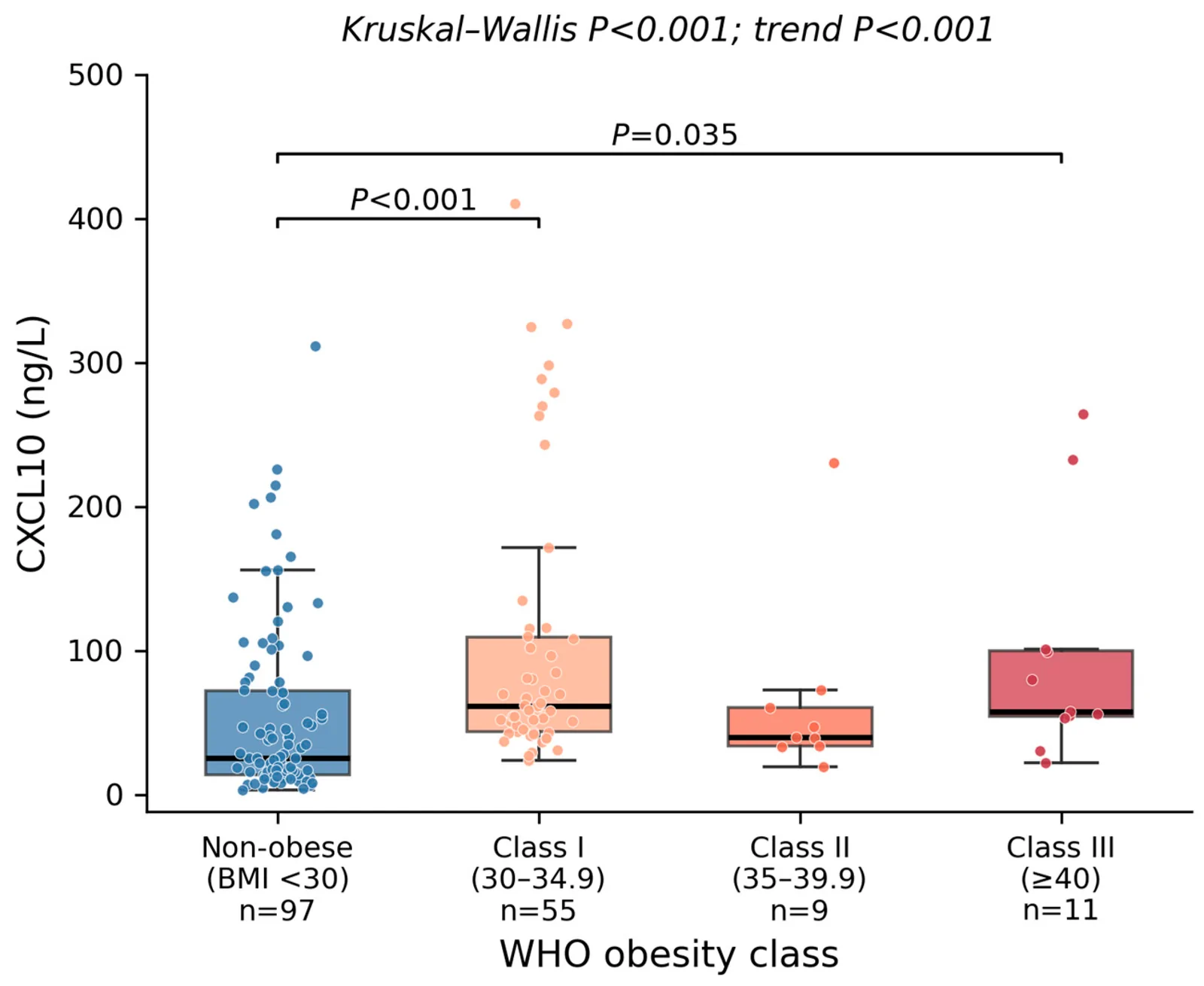
Serum CXCL10 concentrations according to World Health Organization obesity class. Box plots are drawn as in Figure 1. *p* values are from the Kruskal–Wallis test across the four BMI categories and the Jonckheere–Terpstra trend test; brackets show Dunn post hoc comparisons versus the non-obese category with Bonferroni adjustment.

When participants were stratified by World Health Organization obesity class, serum CXCL10 differed across the four BMI categories (*p* < 0.001). An ordered increasing trend was present across the full BMI spectrum (Jonckheere–Terpstra *p* < 0.001; Spearman ρ for ordinal class, 0.38; *p* < 0.001). Median CXCL10 was 25.4 ng/L (IQR, 13.8 to 72.1) in non-obese participants, 61.4 ng/L (IQR, 43.7 to 109.0) in class I, 39.8 ng/L (IQR, 33.6 to 60.5) in class II, and 57.6 ng/L (IQR, 54.0 to 100.1) in class III obesity (Table 4 and Figure 4). Concentrations exceeded the 29.6 ng/L cutoff in 45.4% of non-obese participants and in 88.9–96.4% of participants within each obesity class. In Dunn post hoc comparisons, CXCL10 was higher in class I (adjusted *p* < 0.001) and class III (adjusted *p* = 0.035) than in the non-obese category, whereas the comparison for class II (*n* = 9) did not reach significance. Among the three obesity classes, CXCL10 neither differed (*p* = 0.17) nor showed an ordered trend (*p* = 0.38) and was not correlated with BMI within the obese range (ρ = −0.18, *p* = 0.12). The association between CXCL10 and obesity class persisted after rank-based adjustment for age and sex (partial r = 0.39, *p* < 0.001). Therefore, the elevation relative to non-obese participants was present from class I onwards, without a graded increase across successive obesity classes.

### 3.6. Sex-Stratified and Sensitivity Analyses

**Figure 5:**
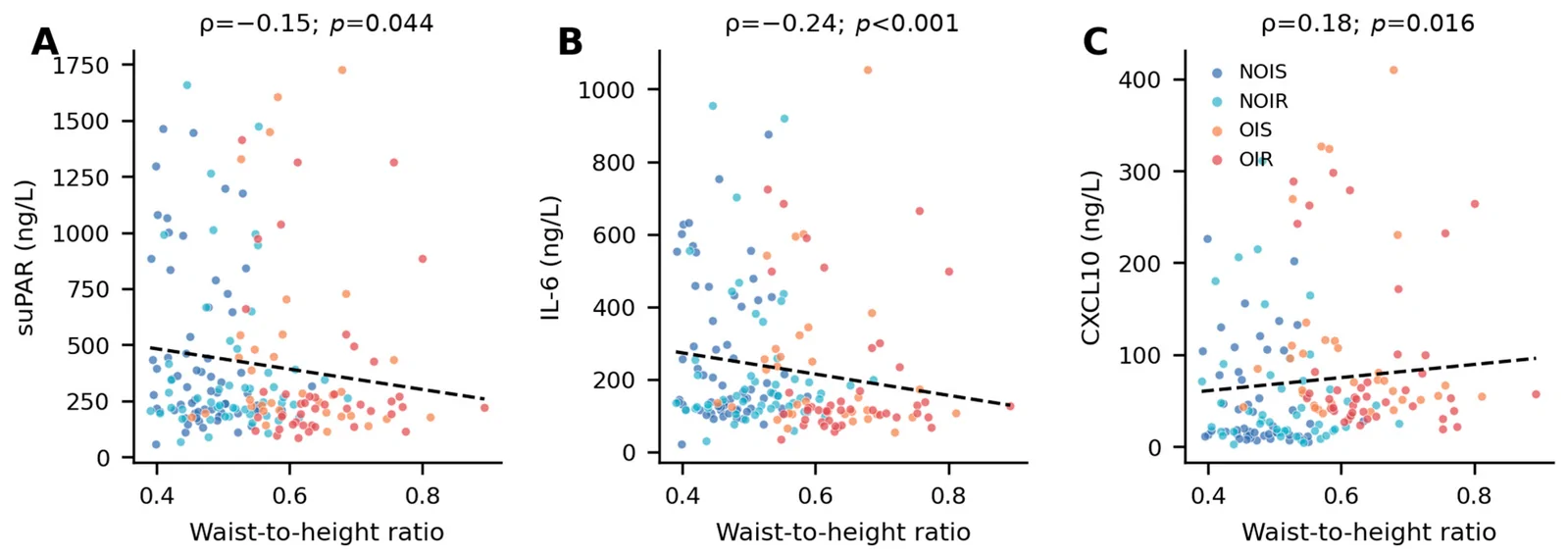
Serum suPAR (Panel (**A**)), IL-6 (Panel (**B**)), and CXCL10 (Panel (**C**)) concentrations in relation to the waist-to-height ratio. Points are coloured by metabolic phenotype, and dashed lines are linear fits. Spearman correlation coefficients and *p* values are shown above each panel.

Serum concentrations of the three biomarkers were similar in women and men (*p* ≥ 0.22 for each), and rank-based models with obesity status and sex as factors showed no main effect of sex and no obesity by sex interaction for any biomarker (*p* ≥ 0.22 and *p* ≥ 0.57, respectively). The elevation of CXCL10 in obesity was present within each sex (Table 5): median concentrations in obese versus non-obese participants were 58.5 versus 26.7 ng/L among women (*p* < 0.001) and 49.2 versus 19.5 ng/L among men (*p* = 0.005), and CXCL10 correlated with BMI to a similar degree in women (ρ = 0.28, *p* = 0.002) and men (ρ = 0.33, *p* = 0.027). In sex-stratified logistic regression adjusted for age and CRP, each 10 ng/L increase in CXCL10 remained associated with obesity among women (odds ratio, 1.12; 95% CI, 1.05 to 1.19) and among men (odds ratio, 1.18; 95% CI, 1.01 to 1.37). suPAR did not differ across the four phenotypes in either sex (*p* = 0.42 in women; *p* = 0.35 in men). In sensitivity analyses with HOMA-IR cutoffs of 2.0 and 3.0, the main effect of obesity on CXCL10 persisted (*p* < 0.001 at each cutoff), whereas no main effect of insulin resistance emerged for any biomarker at any cutoff (*p* ≥ 0.13) (Table 5). When HOMA-IR was examined as a continuous variable, none of the biomarkers correlated with it (ρ range, −0.11 to 0.05; *p* ≥ 0.13), and the corresponding rank-based partial correlations adjusted for BMI, age, and sex were also null (*p* ≥ 0.40). In line with these results, the associations of the three biomarkers with the waist-to-height ratio followed the same direction as their associations with BMI (Figure 5).

## 4. Discussion

The main finding of the present study was that among the three inflammatory mediators examined, CXCL10 separated metabolic obesity phenotypes, and it did so along the axis of adiposity rather than insulin resistance. Serum suPAR neither differed across phenotypes nor correlated with HOMA-IR, and IL-6 was, unexpectedly, lowest in the obese insulin-resistant group. CRP, measured in routine care, behaved as anticipated and rose stepwise with phenotype severity, which indicates that the expected metabolic gradient was present in the cohort.

The CXCL10 findings extend prior tissue-level observations to the circulation. Adipose tissue expression of CXCL10 is increased in obesity and correlates with BMI [14], and human adipocytes secrete CXCL10 constitutively and upon interferon stimulation [13]. Our data indicate that this adipose-associated signal is detectable in the serum and is proportional to fat mass: the correlation with BMI persisted after adjustment for HOMA-IR, age, and sex, and each 10 ng/L increment conferred 12% higher odds of obesity independently of CRP. The absence of any association between serum CXCL10 and HOMA-IR contrasts with the findings of Kochumon et al., in whose cohort adipose CXCL chemokine expression accompanied markers of metabolic inflammation and the related chemokine CXCL11 tracked HOMA-IR [14]. An explanation for this may be compartmentalisation, as chemokine concentrations within adipose tissue could mirror local immune cell recruitment associated with insulin signalling, whereas the circulating pool contains contributions from multiple tissues and may therefore dilute signals specific to a particular depot [12]. In addition, the stringent exclusion criteria applied in the present study, in particular the exclusion of participants with CRP levels above 5 mg/L, may have removed individuals with more pronounced systemic inflammation and thereby attenuated any relation between circulating CXCL10 and metabolic inflammatory markers. At the discrimination level, CXCL10 performed no better than CRP; therefore, the practical value of the marker in this context appears descriptive rather than diagnostic.

Stratification by obesity class refined the adiposity signal. CXCL10 increased in an ordered fashion across the full BMI spectrum. Within the obese range, however, the concentrations reached in class I obesity were not exceeded in classes II and III, and CXCL10 was unrelated to BMI among obese participants. This pattern suggests a threshold rather than a graded relationship between fat mass and the circulating chemokine. Population data are compatible with such a trajectory: among more than 1700 adults in the PLCO screening trial, serum CXCL10 rose with each 5 kg/m^2^ increment of BMI, largely across the transition from normal weight to obesity [20]. At the upper end of the scale, Schinner et al. found no further increase in circulating inflammatory mediators within morbid obesity, despite deteriorating glucose tolerance [21]. This observation accords with a saturable inflammatory output of expanded adipose tissue. Because classes II and III comprised few participants (*n* = 9 and *n* = 11, respectively) in the present cohort, the apparent plateau requires confirmation in larger samples before firm conclusions can be drawn. If confirmed, it would reinforce the interpretation that circulating CXCL10 marks the presence of expanded fat mass rather than scaling with its degree, in keeping with the similar concentrations observed in the OIS and OIR phenotypes.

The null result for suPAR deserves careful interpretation because it contrasts with a substantial body of epidemiological evidence. In Danish general population cohorts, suPAR predicted incident T2DM, cardiovascular disease, and mortality [8,9], and paediatric case–control data reported higher suPAR in adolescents with obesity [19]. Two factors might explain the differences between our observations and those of these reports. First, the discordance is smaller than it appears: Heraclides et al. found that the association between suPAR and incident T2DM was confined to overweight participants and disappeared in obese participants [10], and population-based data have linked suPAR to adiposity mainly at the extreme of morbid obesity [22]. Our cohort, which excluded diabetes and active inflammation and was comparatively young, may occupy exactly the range in which the suPAR signal is weakest. Second, absolute suPAR concentrations in the present study (medians of 225 to 292 ng/L, corresponding to 0.23 to 0.29 ng/mL) were roughly an order of magnitude lower than the 2 to 4 ng/mL range reported in healthy adults with widely used assays [11,22]. Calibration differences between ELISA kits are well documented for suPAR [11]. The roughly tenfold discrepancy in absolute values more plausibly reflects the assay than the study population. suPAR circulates as full-length and cleaved forms [6], and the antibody pairs of different kits may recognise these forms to varying extents [7]. Because the present kit was not calibrated against these widely used platforms, its absolute values are interpretable only within this dataset and cannot be placed on the scale of published reference intervals or cutoffs. If the kit compresses true differences, the effect would be to reduce power and favour a null result. Compression of this kind would not, however, explain the weak inverse correlations of suPAR with BMI and CRP. These run counter to the positive correlation between suPAR and CRP in the general population [8] and are difficult to reconcile with a biological explanation; a more likely source is discussed below. Under these assay conditions, the prognostic value of suPAR reported in cohorts with standardised assays, including its association with mortality in T2DM [23], cannot be excluded by our data; what our data do not support is the use of the present assay configuration to separate insulin-resistant from insulin-sensitive individuals.

The correlation structure of the three ELISA-measured markers also requires comment. The markers were strongly intercorrelated (ρ 0.57 to 0.87), overall and within each phenotype. None, however, correlated positively with CRP, which was measured independently in the hospital laboratory. Modest positive correlations among inflammatory markers are usual [7]. Three mediators with different cellular sources and regulation would nevertheless not be expected to covary as closely as observed here, and a factor common to all three measurements is the most parsimonious explanation. Such a factor is plausible, because all three were measured on aliquots of the same serum sample with kits from the same manufacturer after an identical storage interval. Matrix effects, storage time, or plate position could then generate intercorrelation without biological meaning. Because the intercorrelations were similar within each phenotype, a shared component unrelated to phenotype would mainly inflate covariation and reduce power. A sample-related factor that itself varied with adiposity, such as a matrix effect, could in addition impart weak associations of the same direction to all three markers. Such an effect cannot be excluded for suPAR and IL-6. We therefore draw no conclusion about a coordinated inflammatory response from these correlations. The CXCL10 finding is less vulnerable to this concern. It alone showed a positive association with adiposity, consistent with the tissue-level data cited above, which persisted after adjustment for independently measured CRP and was consistent in both sexes. A technical factor common to all three assays would not be expected to produce a positive signal confined to one analyte. Replication with assays validated against reference methods is nonetheless required before the suPAR and IL-6 patterns can be interpreted biologically.

The inverse relationship between IL-6 and adiposity was unexpected, given the established contribution of adipose tissue to circulating IL-6. Several explanations merit consideration. IL-6 is a pleiotropic cytokine with context-dependent metabolic actions, and circulating concentrations respond to muscle activity and sampling conditions in ways that a single fasting measurement cannot capture [24]. Three features of the data, however, argue against a biological explanation. First, the effect was modest: the only significant post hoc contrast was OIR versus NOIS, and the correlations with adiposity indices were weak. Second, the difference was present in women but not in the small male subgroup. Third, IL-6 was very strongly correlated with suPAR (ρ = 0.87) and shared its weak inverse relations with BMI, the waist-to-height ratio, and CRP. The inverse IL-6 pattern is therefore not independent of the suPAR result and is more plausibly assay-related than a true departure from the reported rise of circulating IL-6 with fat mass [15].

Taken together, the results address the central question of the study: whether the inflammatory burden of obesity follows fat mass or metabolic dysfunction. For circulating CXCL10, the answer in this cohort was fat mass, since metabolically healthy and unhealthy obese participants had similar concentrations, a finding consistent with the view that some inflammatory features of obesity are present even in the metabolically healthy phenotype [3,4]. The consistency of this pattern in both sexes and across alternative HOMA-IR cutoffs argues against an explanation based on the predominance of women in the sample or on misclassification near the 2.5 threshold. Follow-up of such cohorts appears warranted because metabolically healthy obesity often converts to an unhealthy phenotype over time, and its cardiovascular risk is not neutral [5].

## 5. Limitations

Several limitations of the present study should be acknowledged. The cross-sectional, single-center design precluded causal inference and limited generalisability. Insulin resistance was estimated using HOMA-IR rather than clamp techniques, and misclassification near the 2.5 cutoff is possible. Absolute biomarker concentrations depend on the specific ELISA kits used; the low suPAR values relative to published reference ranges suggest calibration differences that hamper comparison with other cohorts, and the strong intercorrelation of the three assays may reflect shared measurement variance. This possibility cannot be separated from true biological covariation in the present dataset, and the inverse patterns observed for suPAR and IL-6 should therefore be interpreted with particular caution. Obese participants were older than non-obese participants; although adjusted analyses addressed this imbalance, residual confounding cannot be excluded. Smoking status and body composition measures were not available, and women constituted approximately three-quarters of the sample, which may limit applicability to men. Although sex-stratified analyses yielded concordant results, the male subgroup was small, and the corresponding estimates were imprecise. In addition, the small number of participants with class II and class III obesity restricts the precision of the analyses stratified by obesity severity. The sample size assessment applied to the primary four-group comparison did not extend to these severity-stratified analyses. Furthermore, the analytical performance characteristics of the ELISA assays, including the intra-assay and inter-assay coefficients of variation, are manufacturer-reported values that could not be independently verified in our laboratory, and no reference-method comparison or spike-recovery experiments were performed. Moreover each biomarker was measured at a single time point; therefore, within-person biological variability could not be assessed. Finally, beyond the Bonferroni-adjusted post hoc procedures, no formal correction for multiplicity was applied across the secondary analyses, which should therefore be regarded as exploratory.

## 6. Conclusions

In adults stratified by BMI and HOMA-IR into four metabolic obesity phenotypes, serum CXCL10 was elevated in obesity, irrespective of insulin resistance status, and was independently associated with adiposity, whereas suPAR and IL-6 did not distinguish the phenotypes. These findings identify circulating CXCL10 as an adiposity-linked inflammatory signal, do not support suPAR as an obesity-independent marker of insulin resistance under the present assay conditions, and underline the need for assay harmonisation in inflammation biomarker research. Prospective studies with standardised measurements should test whether phenotype-specific inflammatory profiles improve metabolic risk stratification beyond BMI and CRP levels.

## Figures and Tables

**Table 1:** Baseline characteristics of the study population according to metabolic obesity phenotype.

Characteristic	NOIS (*n* = 64)	NOIR (*n* = 50)	OIS (*n* = 33)	OIR (*n* = 43)	*p* Value †
Age, yr	24.0 (22.0–34.3)	25.0 (22.0–36.0)	33.0 (27.0–43.0)	32.0 (24.0–43.5)	0.005
Female sex, *n* (%)	47 (73.4)	35 (70.0)	25 (75.8)	31 (72.1)	0.95
Body-mass index, kg/m^2^	22.5 (20.7–24.2)	24.7 (22.9–26.8)	31.2 (30.4–34.0)	33.6 (30.7–36.2)	<0.001
Waist circumference, cm	77.0 (71.0–86.0)	87.0 (76.3–97.0)	102.0 (91.0–112.0)	106.0 (98.0–113.5)	<0.001
Fasting glucose, mg/dL	88.0 (83.8–92.3)	96.0 (90.0–100.0)	87.0 (83.0–92.0)	95.0 (90.5–102.5)	<0.001
Fasting insulin, µU/mL	7.4 (5.9–9.6)	14.0 (12.3–17.3)	9.0 (7.8–10.8)	18.8 (14.5–23.9)	<0.001
HOMA-IR	1.66 (1.31–2.06)	3.35 (2.82–4.21)	2.03 (1.65–2.36)	4.08 (3.35–5.74)	<0.001
HbA1c, %	5.1 (5.0–5.3)	5.3 (5.2–5.5)	5.3 (5.0–5.4)	5.4 (5.1–5.9)	<0.001
Total cholesterol, mg/dL	164 (141–191)	171 (156–193)	179 (155–198)	182 (164–205)	0.062
LDL cholesterol, mg/dL	89 (70–114)	97 (84–117)	113 (87–125)	109 (95–130)	0.007
HDL cholesterol, mg/dL	57 (48–64)	49 (42–61)	49 (43–59)	47 (38–52)	<0.001
Triglycerides, mg/dL	69 (55–95)	86 (72–126)	101 (71–119)	128 (97–160)	<0.001
Uric acid, mg/dL	4.4 (3.7–5.2)	5.0 (4.2–5.6)	5.0 (4.1–5.3)	5.3 (4.5–6.7)	0.020
ALT, U/L	12.4 (10.0–16.3)	16.5 (11.4–28.5)	16.0 (11.8–21.3)	20.0 (14.5–24.5)	<0.001
CRP, mg/L	1.0 (1.0–1.6)	1.2 (1.0–2.9)	2.0 (1.0–3.0)	3.0 (2.0–4.0)	<0.001
White blood cells, ×10^3^/µL	6.29 (5.54–7.33)	7.55 (6.71–8.38)	7.57 (6.15–9.04)	7.14 (6.64–8.00)	<0.001

Values are medians (interquartile ranges) unless otherwise indicated. † *p* values are from Kruskal–Wallis tests for continuous variables and the chi-square test for sex. ALT, alanine aminotransferase; CRP, C-reactive protein; HbA1c, glycated haemoglobin; HDL, high-density lipoprotein; HOMA-IR, homeostasis model assessment of insulin resistance; LDL, low-density lipoprotein; NOIR, non-obese insulin-resistant; NOIS, non-obese insulin-sensitive; OIR, obese insulin-resistant; OIS, obese insulin-sensitive.

**Table 2:** Serum inflammatory biomarker concentrations according to metabolic obesity phenotype.

Biomarker	NOIS (*n* = 64)	NOIR (*n* = 50)	OIS (*n* = 33)	OIR (*n* = 43)	*p* Value †	Obesity Effect ‡	IR Effect ‡
suPAR, ng/L	292 (205–565)	255 (197–388)	258 (195–481)	225 (152–288)	0.19	0.24	0.13
IL-6, ng/L	154 (119–381)	152 (122–212)	135 (105–263)	116 (92–166) §	0.016	0.008	0.18
CXCL10, ng/L	19.4 (12.6–77.0)	28.9 (17.4–67.2)	70.1 (51.0–110.1) ‖	53.1 (38.7–80.9) ‖	<0.001	<0.001	0.71

Values are medians (interquartile ranges). † Kruskal–Wallis test across the four phenotypes. ‡ Main effects from the rank-based two-factor (obesity × insulin resistance) Scheirer–Ray–Hare analysis; the interaction term was not significant for any of the three biomarkers. § Adjusted *p* = 0.012 versus NOIS in Dunn post hoc comparison. ‖ Adjusted *p* ≤ 0.034 versus both NOIS and NOIR in Dunn post hoc comparisons. CXCL10 denotes C-X-C motif chemokine ligand 10, IL-6 interleukin-6, IR insulin resistance, and suPAR soluble urokinase plasminogen activator receptor; phenotype abbreviations as in Table 1.

**Table 3:** Performance of inflammatory biomarkers for the identification of obesity and insulin resistance.

Analysis	Marker	AUC (95% CI)	Cutoff	Sensitivity, %	Specificity, %
Obesity (BMI ≥ 30 kg/m^2^)	CXCL10	0.74 (0.66–0.81)	29.6 ng/L	94.7	54.6
	CRP	0.72 (0.65–0.80)	1.2 mg/L	81.6	61.4
Insulin resistance (HOMA-IR ≥ 2.5)	suPAR	0.43 (0.35–0.51)	—	—	—
	IL-6	0.43 (0.35–0.51)	—	—	—
	CXCL10	0.50 (0.42–0.59)	—	—	—

AUC denotes the area under the receiver operating characteristic curve and CI denotes the confidence interval. Cutoffs were derived using the Youden index; cutoffs are not reported where the AUC did not indicate discriminative ability.

**Table 4:** Serum CXCL10 concentrations according to World Health Organization obesity class.

BMI Category	No. of Participants	BMI, kg/m^2^	CXCL10, ng/L	CXCL10 ≥ 29.6 ng/L, *n* (%)	Adjusted *p* Value †
Non-obese (<30.0)	97	23.8 (22.3–25.9)	25.4 (13.8–72.1)	44 (45.4)	Reference
Obesity class I (30.0–34.9)	55	30.8 (30.4–33.2)	61.4 (43.7–109.0)	53 (96.4)	<0.001
Obesity class II (35.0–39.9)	9	36.3 (36.1–38.3)	39.8 (33.6–60.5)	8 (88.9)	>0.99
Obesity class III (≥40.0)	11	41.7 (41.5–43.7)	57.6 (54.0–100.1)	10 (90.9)	0.035

Values are medians (interquartile ranges) unless otherwise indicated. *p* < 0.001 across the four BMI categories (Kruskal–Wallis test) and for the ordered trend across categories (Jonckheere–Terpstra test); the three obesity classes did not differ from one another (*p* = 0.17). The 29.6 ng/L threshold is the Youden cutoff for obesity from the ROC analysis. † Dunn post hoc comparison versus the non-obese category with Bonferroni adjustment. BMI denotes body-mass index and CXCL10 C-X-C motif chemokine ligand 10.

**Table 5:** Sex-stratified inflammatory biomarker concentrations and sensitivity analyses for the definition of insulin resistance.

**Biomarker**	**Non-Obese**	**Obese**	***p* Value †**	***p* Value Across Phenotypes ‡**
Women (*n* = 138)				
suPAR, ng/L	280 (206–443)	227 (179–456)	0.16	0.42
IL-6, ng/L	154 (124–291)	119 (96–240)	0.006	0.045
CXCL10, ng/L	26.7 (15.9–78.3)	58.5 (45.4–104.9)	<0.001	<0.001
Men (*n* = 52)				
suPAR, ng/L	255 (194–477)	251 (193–431)	0.83	0.35
IL-6, ng/L	144 (115–284)	121 (105–264)	0.31	0.33
CXCL10, ng/L	19.5 (12.5–60.7)	49.2 (38.2–97.2)	0.005	0.024
Sensitivity Analyses for the Definition of Insulin Resistance §				
**Biomarker**	**HOMA-IR ≥ 2.0**	**HOMA-IR ≥ 2.5**	**HOMA-IR ≥ 3.0**	**Continuous HOMA-IR ‖**
suPAR	0.25/0.31	0.24/0.13	0.30/0.22	ρ = −0.09; *p* = 0.24
IL-6	0.011/0.21	0.008/0.18	0.011/0.42	ρ = −0.11; *p* = 0.13
CXCL10	<0.001/0.39	<0.001/0.71	<0.001/0.38	ρ = 0.05; *p* = 0.51

Values are medians (interquartile ranges); serum CXCL10 was available in 126 women and 46 men. † Mann–Whitney test for obese versus non-obese participants within each sex. ‡ Kruskal–Wallis test across the four metabolic phenotypes within each sex. § Entries are main-effect *p* values (obesity/insulin resistance) from rank-based two-factor Scheirer–Ray–Hare models with insulin resistance defined by the indicated HOMA-IR cutoff; the 2.5 cutoff corresponds to the primary analysis. ‖ Spearman correlation between each biomarker and HOMA-IR analysed as a continuous variable. Phenotype and biomarker abbreviations as in Table 1 and Table 2.

## Data Availability

The data supporting the findings of this study are available from the corresponding author upon reasonable request.
